# Effects of temperature on the transmission of *Yersinia Pestis *by the flea, *Xenopsylla Cheopis*, in the late phase period

**DOI:** 10.1186/1756-3305-4-191

**Published:** 2011-09-29

**Authors:** Anna M Schotthoefer, Scott W Bearden, Jennifer L Holmes, Sara M Vetter, John A Montenieri, Shanna K Williams, Christine B Graham, Michael E Woods, Rebecca J Eisen, Kenneth L Gage

**Affiliations:** 1 Bacterial Diseases Branch, Division of Vector Borne Diseases, National Center for Emerging and Zoonotic, Infectious Diseases, Centers for Disease Control and Prevention, Fort Collins, CO 80521, USA; 2 Marshfield Clinic Research Foundation, 1000 North Oak Avenue, Marshfield, WI 54449, USA; 3 Minnesota Department of Health, P. O. Box 64975, St Paul, MN 55164, USA; 4 Lawrence Livermore National Laboratory, 7000 East Avenue. L-174, Livermore, CA 94550, USA

**Keywords:** *Yersinia pestis*, *Xenopsylla cheopis*, biofilm, flea-borne transmission, temperature

## Abstract

**Background:**

Traditionally, efficient flea-borne transmission of *Yersinia pestis*, the causative agent of plague, was thought to be dependent on a process referred to as blockage in which biofilm-mediated growth of the bacteria physically blocks the flea gut, leading to the regurgitation of contaminated blood into the host. This process was previously shown to be temperature-regulated, with blockage failing at temperatures approaching 30°C; however, the abilities of fleas to transmit infections at different temperatures had not been adequately assessed. We infected colony-reared fleas of *Xenopsylla cheopis *with a wild type strain of *Y. pestis *and maintained them at 10, 23, 27, or 30°C. Naïve mice were exposed to groups of infected fleas beginning on day 7 post-infection (p.i.), and every 3-4 days thereafter until day 14 p.i. for fleas held at 10°C, or 28 days p.i. for fleas held at 23-30°C. Transmission was confirmed using *Y. pestis*-specific antigen or antibody detection assays on mouse tissues.

**Results:**

Although no statistically significant differences in per flea transmission efficiencies were detected between 23 and 30°C, efficiencies were highest for fleas maintained at 23°C and they began to decline at 27 and 30°C by day 21 p.i. These declines coincided with declining median bacterial loads in fleas at 27 and 30°C. Survival and feeding rates of fleas also varied by temperature to suggest fleas at 27 and 30°C would be less likely to sustain transmission than fleas maintained at 23°C. Fleas held at 10°C transmitted *Y. pestis *infections, although flea survival was significantly reduced compared to that of uninfected fleas at this temperature. Median bacterial loads were significantly higher at 10°C than at the other temperatures.

**Conclusions:**

Our results suggest that temperature does not significantly effect the per flea efficiency of *Y. pestis *transmission by *X. cheopis*, but that temperature is likely to influence the dynamics of *Y. pestis *flea-borne transmission, perhaps by affecting persistence of the bacteria in the flea gut or by influencing flea survival. Whether *Y. pestis *biofilm production is important for transmission at different temperatures remains unresolved, although our results support the hypothesis that blockage is not necessary for efficient transmission.

## Background

It is well documented that temperature is an important regulator of the transmission dynamics of vector-borne pathogens. This relationship is largely related to the effects that temperature has on the survival, growth, development, and reproduction of pathogens and their vectors [1,2]. Temperature also has the ability to alter the behavior and activity of vectors, resulting in changes in contact rates between vectors, pathogens, and the hosts that may be involved in pathogen life cycles [3]. Changes in temperatures, therefore, have the potential to shift or expand vector-host-pathogen geographic ranges, modify the seasonality or phenology of infections, and lead to changes in pathogen generation times and transmission rates, thereby altering the burden of disease on a host population. These potential changes are of particular concern with regard to the predictions of climate change and its effects on vector-borne diseases [4,5].

*Yersinia pestis*, the causative agent of plague, is a Gram-negative bacterium that infects primarily rodents, but which may also infect a wide array of other mammalian hosts, including humans. Fleas are employed by the pathogen as vectors, and the majority of transmission events are believed to occur through flea bites [6]. Thus, the success of the bacterium depends, in part, on its ability to adapt and quickly respond to the disparate temperature environments encountered in the mammalian and flea hosts [7]. Success within the flea, specifically, appears to be linked to the ability of *Y. pestis *to rapidly form a biofilm that is based on the synthesis of an extracellular polysaccharide matrix once inside the flea gut lumen [8]. The production of a biofilm creates large aggregates of *Y. pestis*, which presumably are not easily removed from the flea's gut via defecation, and attachment and growth of the biofilm on the surfaces of the proventriculus, a valve that connects the esophagus to the midgut, act to plug the gut lumen, in a condition referred to as blockage. Blockage impedes the ability of the flea to ingest subsequent blood meals, forcing any blood taken up to be regurgitated back into the host. It is believed that this action associated with attempted feedings causes some of the bacteria to be dislodged from the biofilm and injected into the host with the blood [8-10]. Transmission by blocked fleas is the mechanism most frequently recognized as an integral, and possibly essential, component of efficient flea-borne transmission [8].

Evidence suggests that the blockage mechanism of flea transmission is mediated by temperature. The ability of the bacteria to colonize the proventriculus and cause blockage in the flea is dependent on the hemin storage locus (*hms*) gene complex [11,12], and appears to be optimized around 20-26°C [10], the temperature range that would be typical of the flea gut environment in the nests and burrows of rodents in many situations [13,14]. As temperature increases from 26°C, the ability for *Y. pestis *to cause blockage declines and fails and 30°C [15], and hms proteins are degraded circa 37°C, the body temperature of the mammalian host [10,16]. Such observations suggest that biofilm production and flea-borne transmission of *Y. pestis *will decline with rising environmental temperatures, and previous investigations have, in part, supported these predictions. Kartman and Prince [17] reported that fleas held at 20°C had the potential to transmit an average of 1.75 new infections to naïve mice, in contrast to fleas held at 30°C, which only infected on average 0.56 mice. Kartman [18] also observed a more rapid loss of *Y. pestis *infections in fleas held at 29.5°C compared to fleas at 23.5°C.

The effect of temperature on the transmission potential of fleas has been used to explain the variation in seasonal and regional patterns of plague outbreaks. In particular, the dynamics of *Y. pestis *outbreaks in endemic areas typically show a seasonal pattern, with trends of increasing cases being reported as conditions become warmer and more humid, followed by sharp declines in cases once temperatures exceed about 27.5°C and humidity decreases [19-23]. Thus, the declines in cases during seasonal periods of hot weather may be attributed to the poorer flea transmission efficiency associated with reduced *hms*-mediated biofilm production and flea blockage at high temperatures [10,15,24]. However, a recent examination of the effects of temperature on the transmission efficiency of fleas during the early-phase period between 1-4 days post-infection (p.i.), sensu [25], found that *Xenopsylla cheopis*, the Oriental rat flea, held at 30°C transmitted *Y. pestis *just as efficiently to naïve mice as fleas held at 23°C [26]. Nonetheless, those results do not rule out the possibility that the negative effects of high temperatures predicted for flea transmission efficiency occur during the post-early phase period, when biofilm production and blockage formation is expected to be important (e.g., 12-18 days p.i., [20,27-29]). The primary aim of the current study, therefore, was to explore the effects of warming temperatures on the transmission of *Y. pestis *by *X. cheopis *fleas in the post-early-phase period (referred to here as late phase), between days 7 and 28 p.i. We also continued to examine the effect of a cold temperature (10°C) on flea transmission to determine if the absence of transmission observed during the early-phase period by Schotthoefer et al. [26] was an indication of a loss of vector competence by *X. cheopis *fleas at this low temperature or a delay in acquiring competence.

## Results

### Effects of temperature on flea transmission efficiencies

None of the fleas maintained at our three high temperatures (23, 27 and 30°C) and used in our challenges transmitted infections on day 7 p.i.; however, after this time point, fleas at these temperatures were able to transmit infections through to days 21 or 28 p.i. (Table 1). Our observed per flea transmission efficiencies were highest at 23°C. At this temperature, fleas consistently transmitted at per flea efficiencies greater than 12% until day 28 p.i., when the efficiency dropped to about 4% (Table 1). In comparison, transmission efficiencies were low for fleas maintained at 27°C until they rose to 15.4 and 13.5% on days 17 and 21 days p.i., respectively. The per flea transmission efficiencies observed at 30°C were highest on day 10 p.i. and gradually declined thereafter (Table 1). Despite the trend that per flea efficiencies were higher at 23 than at 27 and 30°C, the likelihood of a mouse becoming infected was not significantly associated with the temperature at which fleas were held (χ^2 ^= 2.9, df = 3, p = 0.41).

**Table 1:** Transmission efficiencies of infected fleas held at different experimental temperatures during the late phase period

Temp (°C)	Days p.i.	Flea infection prevalence (%)	Average no. fed, infected fleas per mouse (total in treatment group)	No. of mice infected (exposed)	Percent per flea transmission efficiency (95% CI)
10	7	100	5.0 (35)	3 (7)	9.81 (2.74, 25.74)
	10	97.1	5.8 (35)	1 (6)	2.75 (0.17, 12.75)
	14	75.9	6.8 (54)	3 (8)	6.31 (1.75, 16.31)
23	7	88.6	6.3 (44)	0 (7)	0 (0, 6.60)
	10	95.2	6.0 (42)	4 (7)	12.48 (4.33, 30.48)
	14	92.3	5.6 (39)	4 (7)	14.11 (4.73, 34.11)
	17	100	4.3 (30)	4 (7)	17.42 (6.04, 37.42)
	21	94.1	4.9 (34)	5 (7)	21.77 (8.67, 48.81)
	28	79.2	4.0 (24)	1 (6)	4.27 (0.25, 20.27)
27	7	98.1	6.5 (52)	0 (8)	0 (0, 5.80)
	10	96.9	4.6 (32)	2 (7)	7.04 (1.30, 23.02)
	14	92.3	5.6 (39)	1 (7)	2.52 (0.15, 11.93)
	17	85.7	4.0 (28)	3 (7)	15.41 (4.03, 44.01)
	21	83.8	5.3 (37)	4 (7)	13.50 (4.75, 31.81)
	28	61.9	5.3 (21)	0 (4)	0 (0, 11.67)
30	7	95.0	5.6 (39)	0 (7)	0 (0, 7.46)
	10	100	5.3 (37)	3 (7)	9.47 (2.64, 25.19)
	14	95.4	6.1 (43)	3 (7)	8.30 (2.32, 22.62)
	17	100	4.1 (29)	2 (7)	7.67 (1.42, 24.58)
	21	93.3	5.0 (30)	0 (6)	0 (0, 9.03)
	28	91.2	6.8 (34)	1 (5)	2.72 (0.18, 12.69)

### Effects of temperature on Y. pestis infections in fleas

**Figure 1:**
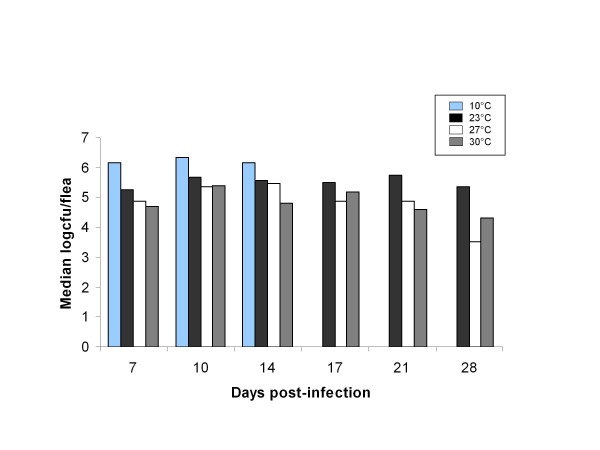
**Effect of temperature on flea bacterial loads**. Median bacterial loads (estimated log_10_[n+1] cfu) per fed flea of fleas exposed to challenge mice at each temperature and time point examined in the experiment.

Infection prevalences, bacterial loads, and flea feeding rates did not differ between male and female fleas maintained at different temperatures (data not shown); therefore, we did not consider this factor in our analyses. Prevalence of infection was influenced by temperature (χ^2 ^= 6.8, df = 2, p = 0.034) and time point (χ^2 ^= 24.0, df = 5, p = 0.0002). Overall prevalences for 23, 27, and 30°C fleas were 91.9, 89.0, and 95.8%, respectively. Declining trends in prevalences were observed over time at 23 and 27°C; however, a significant decline over time was observed only at 27°C (χ^2 ^= 23.9, df = 5, p = 0.0002). Bacterial loads tended to be higher in fleas held at 23°C than in fleas held at 27 and 30°C (Figure 1), although this association was not statistically significant (χ^2 ^= 5.6, df = 2, p = 0.061). Bacterial loads were related to time point (χ^2 ^= 22.8, df = 5, p = 0.0004) and were observed to decline over time in fleas held at 27 and 30°C (Figure 1).

Although the proportions of fleas that successfully took blood meals when exposed to mice in challenge feeds did not vary significantly across temperatures (χ^2 ^= 0.5, df = 2, p = 0.78), they were significantly different across time points at 23°C (χ^2 ^= 12.0, df = 5, p = 0.035) and 27°C (χ^2 ^= 11.6, df = 5, p = 0.040), but not at 30°C (χ^2 ^= 10.5, df = 5, p = 0.063). Feeding rates declined with time at 23 and 27°C with overall feeding rates averaging 64.8% on day 7 p.i. and 46.9% on day 28 p.i. for these temperatures. Fleas maintained at 30°C, had average feeding rates of 55.7% on day 7 p.i. and 68.0% on day 28 p.i.

### Effects of temperature on flea survival

**Figure 2:**
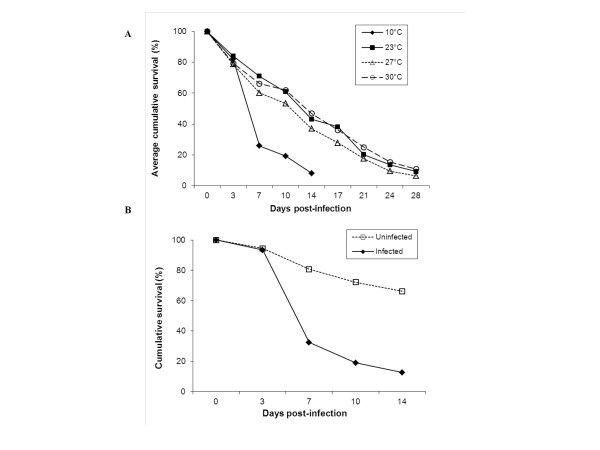
**Effect of temperature on flea survival**. A) Cumulative survival of infected fleas maintained at each temperature during the late phase infection period. Averages were calculated for the flea cohorts used in 12 artificial feeding trials. B) Cumulative survival of fleas used in a separate experiment to examine the effects of *Y. pestis *infection at 10°C.

The proportions of fleas surviving between time points was significantly lower at 27°C compared to 23°C (t value = -2.1, df = 1, p = 0.034) (Figure 2A) and lower on days 14, 21, and 24 p.i. than on day 3 p.i. (all p ≤ 0.036). On day 3 p.i., 84.0% of the fleas maintained at 23°C had survived since obtaining the infectious blood meal on day 0, whereas only 78.7 and 79.4% of the fleas at 27 and 30°C, respectively, had survived. Survival between days 17 and 21 p.i., had declined to 71.2, 67.2, and 68.0% for 23, 27, and 30°C, respectively.

### Predictors of Y. pestis transmission

A stepwise logistic regression analysis incorporating all temperature and time point data identified time point (χ^2 ^= 12.8, df = 5, p = 0.026) and feeding by at least one flea that harbored at least 10^6 ^bacteria (χ^2 ^= 4.01, df = 1, p = 0.045) as predictive of *Y. pestis *transmission to naïve mice. Transmission to mice on day 7 p.i. had a lower probability, whereas the probabilities of transmission on days 17 and 21 p.i were elevated. Mice that were fed on by at least one flea with 10^6 ^bacteria were 2.33 (95% CI: 1.019 - 5.33) times more likely to become infected than mice not fed on by such fleas.

### Transmission of Y. pestis by fleas held at 10°C

Fleas maintained at 10°C, were able to transmit *Y. pestis *to naïve mice on all the days examined in our current study. The per flea transmission efficiencies observed at this temperature were comparable to those observed for 27 and 30°C during the late phase period (Table 1). The bacterial loads in these fleas were maintained on average at a log higher concentration than in the fleas at the three higher temperatures (Figure 1). However, prevalence of infection in the fleas held at 10°C dropped from 100% on day 7 p.i. to 75.9% on day 14 p.i. Feeding rates were not significantly different across time points for the 10°C fleas (χ^2 ^= 4.7, df = 2, p = 0.093). The proportions of fleas surviving between time periods were significantly lower at 10°C compared to the fleas at higher temperatures (χ^2 ^= 459.2, df = 3, p < 0.0001; Figure 2A). In examining the effect of *Y. pestis *infection on flea survival in a separate experiment, we determined that the poor survival was probably due to infection and not simply the cold temperature; fleas infected with *Y. pestis *had higher mortality rates than uninfected fleas at 10°C (t value = 3.4, df = 1, p = 0.042), (Figure 2B). Specifically, infected fleas at days 7, 10, and 14 p.i. were less likely to survive between time periods than uninfected fleas (all ps < 0.0001).

## Discussion

### Effects of high temperatures on Y. pestis transmission

Temperature has long been associated with human and animal plague outbreaks [4,20,30]. Most seasonal outbreaks occur when temperatures range between 24 and 27°C, and declines in the numbers of cases have generally been reported when temperatures exceed a threshold temperature around 27.5°C [20-23,31]. A poor ability of fleas to become blocked, and therefore, transmit infections at high temperatures has been hypothesized as an explanation for these declines [10,15,18,24]. The results from our experimental study partially contradict these predictions because the differences in per flea transmission efficiencies were not statistically significant between 23 and 30°C. In fact, fleas held at 27 and 30°C were reliable transmitters of *Y. pestis *out to at least day 17 p.i., and were still capable of transmitting *Y. pestis *at 21 and 28 days p.i., respectively, even in the absence of blockage. However, we did observe lower transmission efficiencies and flea survival and bacterial loads that declined with time in fleas maintained at 27 and 30°C, suggesting that plague outbreaks would be terminated or at least be of shorter duration at temperatures of 27 and 30°C, as predicted. This was in contrast to fleas held at 23°C which were able to maintain high average per flea transmission efficiencies through day 21 p.i.

The ability for the 23°C fleas to sustain high transmission efficiencies may have been related to their ability to maintain high bacterial loads throughout the late phase period. We found that *Y. pestis *transmission was positively associated with mice that were fed on by at least one flea with a bacterial load of at least 10^6^, and fleas held at 23°C were generally able to retain such high loads longer than fleas held at 27 and 30°C (Figure 1). The ability of *X. cheopis *to maintain high bacterial loads for long periods of time has been previously observed, and has been suggested as a factor contributing to its success as a vector of *Y. pestis*, possibly because fleas with higher bacterial loads are more likely to become blocked [27,32-34]. It should be noted, however, that the early-phase experiments conducted to date have failed to demonstrate a relationship between bacterial load and transmission success [25,26,35-37]. Even in the present study, although mice that became infected were on average fed on by fleas with higher bacterial loads than mice that did not become infected, a flea group with a summed bacterial load as low as 10^4.64 ^was able to transmit an infection, and 13 of our 44 (29.5%) transmission events occurred by flea groups that did not include a flea harboring at least 10^6 ^bacteria. Moreover, 50 of the 81 (61.7%) flea groups that did contain at least one flea with greater than 10^6 ^bacteria failed to transmit *Y. pestis*. Therefore, although mice that became infected were likely to have been fed on by fleas with high bacterial loads, a high flea bacterial load alone was not sufficient for transmission.

The location of bacteria in the flea gut is another factor that may help explain the differences observed in the transmission efficiencies between 23°C and the two higher temperatures. Colonization of the proventriculus by *Y. pestis *and the subsequent production of biofilm by this bacterium have often been cited as necessary for efficient plague transmission because these steps are thought to be prerequisites for blockage [9,27,38]. *Y. pestis *is known to readily colonize the proventriculus and cause blockage in *X. cheopis *at temperatures around 23°C [15,27]. The ability to cause blockage, however, is lost as temperatures approach 30°C [15,18]. It is not clear if the ability for *Y. pestis *to colonize the proventriculus also wanes at 30°C [but see 18], although fleas at 30°C are able to maintain large bacterial masses in their midguts [15]. In addition, temperature-mediated regulation of the *hms *genes, which are required for blockage [38], occurs at the posttranscriptional level, with *Y. pestis *colonies displaying some ability to store hemin, the phenotype often used to assess *hms *activity *in vitro*, at temperatures as high as 35°C [16]. Therefore, it is quite likely that *hms*-mediated biofilm production was not completely inactivated in our study, possibly explaining the ability of fleas held at 27 and 30°C to transmit infections, although the higher transmission efficiencies observed at 23°C may have been related to more bacteria becoming established in the proventriculus than at 27 and 30°C. Future studies should examine whether *Y. pestis *growth occurs in the proventriculus and midgut at 23°C, but primarily occurs only in the midgut in fleas at 30°C, and if colonization of the proventriculus, but not necessarily blockage, of the flea gut is associated with transmission. Moreover, the role of *hms*-mediated biofilm production in transmission at different temperatures remains unresolved.

It is unclear what would explain the lack of transmission by fleas held at 23, 27, or 30°C on day 7 p.i., given that fleas at these temperatures transmitted *Y. pestis *to naïve mice during the early-phase period of infection (1-4 days, p.i.) [26], as well as on later days p.i. in this study, but the observation may also relate to the pattern of growth of *Y. pestis *in the flea gut. Although bacterial masses may begin to form in the proventriculus and esophagus of a flea within the first few days of infection [39,40], complete blockage is not typically observed in *X. cheopis *until 12-18 days p.i. [20,27,29,41]. Day 7 p.i. represents a time point between early-phase and later periods when bacterial masses may be forming in the flea gut, but have not yet reached sufficient mass to disrupt passage of blood to the midgut. Of note, also is the observation that in our experiment, day 7 p.i. fleas represented the first cohort of fleas used in challenge feeds after having their first non-infectious maintenance blood meal on day 3 p.i. It may be that ingestion of a non-infectious blood meal during the period when bacterial masses were just beginning to form (e.g., on day 3 p.i.) acted to flush some of the bacteria from the flea gut or displaced them further back in the midgut, such that bacteria were not as readily regurgitated into mice while they fed on day 7 p.i. Over time, although the total population density of bacteria residing in the flea may be lower, the bacterial masses associated with developing *Y. pestis *colonies and the biofilm they produce may become more persistent and available for transmission during subsequent flea feeding events.

### Effects of 10°C on Y. pestis transmission

Although we reported in an earlier study that *X. cheopis *fleas maintained at 10°C were unable to transmit *Y. pestis *infections to mice during the early-phase period [26], fleas maintained at this temperature were able to transmit *Y. pestis *in the late phase period in our present study, which is consistent with reports by Kartman and Prince [17]. However, our collective observations suggest that *Y. pestis *may behave differently in fleas held under cold environmental conditions. In addition to a delay in the ability to transmit *Y. pestis*, bacterial loads were consistently higher in fleas maintained at 10°C compared to those held at the three warmer temperatures, and these heavy infections at 10°C clearly had a detrimental effect on flea survival, with only about 10% of infected fleas surviving to day 14 p.i. versus about 67% of uninfected fleas. Of interest, also is the observation that both the first transmission event and high flea mortality occurred only after fleas were offered their first maintenance feed on day 3 p.i., suggesting that transmission and mortality were dependent upon obtaining another blood meal following infection.

The differences observed at 10°C may relate to differences in the expression of *Y. pestis *genes at cold versus warm temperatures [42], and to changes in lipopolysaccharide (LPS) structure that occur at cold temperatures [42-45]. For instance, it is possible that the upregulation of *hms *and the yersina murine toxin (*ymt*) gene, which is necessary for *Y. pestis *survival in the flea gut, allows for the extensive bacterial growth observed in the flea at 10°C [42]. It is also possible that the LPS and extracellular matrix that forms at low temperatures may be more resistant to digestion, thus decreasing the chances that bacteria will be dislodged and transmitted during the early-phase period. However, if components of the host blood meal act to digest the *Y. pestis *biofilm (e.g., [46]) then over time, the biofilm may begin to break-up, providing aggregates that may be subsequently transmitted or defecated. Larger aggregates may become dislodged when bacterial loads are exceptionally high and interfere with the ability of fleas to digest blood or defecate, causing the high mortality observed at 10°C. It is also possible that components of the biofilm are directly more toxic to fleas at 10°C [15].

### General implications for plague transmission

Although we have demonstrated that the ability of *X. choepis *fleas to transmit *Y. pestis *is not significantly impaired by high temperatures, our overall results suggest that plague outbreaks would be less likely to be maintained at 27 and 30°C than at 23°C. Bacterial loads declined in the fleas held at 27 and 30°C and fleas held at 27°C experienced higher mortality than fleas at 23°C. It is unclear if these deficiencies in the fleas at the higher temperatures can help explain the declines in human and animal cases of plague observed during hot weather. It is possible that flea survival is simply compromised during hot, dry weather [1,18,21,33]. Fleas expend more energy at higher temperatures [47], and therefore, may be more susceptible to starvation or dehydration under such conditions, such that flea densities and flea-host contact rates are not sufficient to sustain epizootic activity. Flea physiological responses at different temperatures, in addition to the *Y. pestis *responses we've discussed, are also likely to play important roles in the abilities of fleas to maintain or clear *Y. pestis *infections. Moreover, the fed status of the flea and the source of blood meal are other factors that may contribute to the energy demands and survival of fleas, as well as the maintenance of *Y. pestis *infections [46-48]. To better understand plague transmission dynamics, a shift in focus on the effects of temperature on these flea-related factors rather than on blockage in the flea may be warranted given our results and recent modeling efforts that have demonstrated the potential significance of unblocked fleas in driving plague dynamics [49].

## Conclusions

Our results emphasize the conclusions that have been made by previous investigators that flea-borne transmission of plague relates to several factors that have been described in the context of the vectorial capacity model: the likelihood of fleas becoming infectious, the ability of fleas to transmit infections once infectious, and the longevity of fleas once infectious [41,50-52]. Another factor that is likely important is the ability of fleas to retain infections [37,46]. We have shown here that temperature has the potential to modify all of these factors, but that high temperatures alone do not appear to significantly impair the ability of fleas to transmit *Y. pestis *infections. Our work does, however, question how we define an infectious flea.

In their seminal work, Bacot and Martin [9] described flea infectiousness as it related to the formation of proventricular blockage by *Y. pestis*. Their flea blockage model, subsequently, came to dominate the transmission experiments that followed, even though these authors stressed that partially blocked fleas might also be efficient transmitters and would survive longer than blocked fleas and, thus, would have more opportunities to transmit plague to their rodent hosts [53]. In recent years, evidence has accumulated that supports the concept that partially blocked or unblocked fleas are capable of efficiently transmitting *Y. pestis *under certain conditions. In particular, the demonstration that fleas may become infectious within 24 hours or less following ingestion of an infected blood meal in the early-phase transmission studies by Eisen and others [25,26,35,36,54] strongly challenges the assumption that blocked fleas are necessary for efficient transmission. Observing efficient transmission of *Y. pestis *by fleas maintained at 30°C, a temperature in which blockage is not expected [15,24], in the current study raises additional questions regarding the requirement for blockage. The mechanism by which unblocked fleas transmit infections is unclear, but may involve a similar process to that described for blocked fleas in that bacteria get dislodged from a bacterial mass and injected into a host with regurgitated blood; whether this process is more likely if the bacteria have colonized the proventriculus, and how temperature may influence this colonization, remains unanswered.

In conclusion, our results suggest that what defines a flea as an efficient transmitter of plague should be reexamined and that the regulation of biofilm production in the flea under varying environmental conditions should be further studied to understand how transmission efficiency depends on this process. Such studies would improve our ability to adequately assess and make predictions regarding the effects of climate change on *Y. pestis*' life cycle and transmission dynamics.

## Methods

### Infection of fleas

Methods for infecting fleas, confirming transmission of *Y. pestis *from fleas to naïve, 6-wk-old female Swiss Webster (SW) or SW/CD-1 hybrid outbred mice, quantifying bacterial loads in fleas, and evaluating vector competency were similar to those described by Eisen et al. [25] and Schotthoefer et al. [26]. Briefly, male and female adult fleas of mixed ages were randomly removed from an established colony of *X. cheopis *and starved for 4-7 days at 23°C. Twenty-four hours prior to being offered an infectious blood meal, fleas were randomly assigned to one of the four temperature treatment groups: 10, 23, 27, or 30°C, placed in glass bell jars in which the relative humidity was maintained around 85% with a saturated potassium chloride solution [55], and put into incubators (Model 3960 Forma Environmental Chamber, Thermo Scientific, Asheville, NC) set at the corresponding temperatures.

An artificial feeding system, previously described [25] and the fully virulent *Y. pestis *strain, CO963188 [25,26,35,36,54] were used in the current study to infect fleas. On day 0, fleas were removed from incubators and placed in artificial feeders containing on average (±1 s.d.) 1.69 × 10^9 ^(± 1.729 × 10^9^) colony-forming units (cfu)/ml of *Y. pestis *CO9631880 in defibrinated Sprague-Dawley rat blood pre-warmed to 37°C. After 1 hr, fleas were removed from feeders and examined using light microscopy. Fleas that had red blood in their guts were identified as having fed and were considered infected and were placed back into incubators and maintained at their respective treatment temperatures; any fleas that did not take a potentially infectious blood meal were discarded.

### Flea-borne transmission to naïve mice

To assess the transmission efficiency of fleas held at different temperatures in the late phase period, groups of 10 fleas each were removed from incubators and placed on anesthetized naïve mice at room temperature for 1 hr. At the three higher temperatures: 23, 27, 30°C, transmission efficiency was assessed on days 7, 10, 14, 17, 21, and 28 post-infection (p.i.), but because of poor flea survival at 10°C, we were only able to test the efficiency at this temperature on days 7, 10, and 14 p.i. Following exposure to the naïve mice, the fed status and sex of each flea used in the challenges were determined, and they were placed individually in microcentrifuge tubes and stored at -70°C. The remaining surviving fleas not used for a given time point challenge were offered maintenance blood meals on naïve mice at room temperature for 1 hr, beginning on day 3 p.i. and every 3-4 days thereafter until the final time point, and then returned to the incubators. This was done to help promote survival of the fleas.

Mice used in the challenge feeds were subsequently held in separate filter-top cages, observed daily, and euthanized at the onset of symptoms considered indicative of *Y. pestis*-induced illness (e.g., slow response to stimuli, ruffled fur). Successful transmission was confirmed in these ill mice by *Y. pestis *anti-F1 antigen direct fluorescent antibody assays (DFA) of liver and spleen smears. Mice that did not display plague symptoms were euthanized on day 21 p.i., and the serum collected from their blood was tested for evidence of resolved infections using passive hemagglutination and inhibition tests (PHA/HI) for antibodies to *Y. pestis *F1 antigen [56]. A total of 1408 fleas from 12 artificial feeding events were used to obtain a minimum of 4 replicates (= challenged mice) for each temperature and time point. No detectable differences in transmission patterns were observed between SW and SW/CD-1 outbred mice or the fleas used from different artificial feeds (data not shown).

We did not attempt to quantify the proportion of blocked fleas in our study because we felt our attempts to determine the blockage status of fleas using microscopic examinations were too subjective. Moreover, blocked fleas have been observed to become unblocked during a feeding event [34,40,57], and partial blockages, which have been speculated to be as important in transmission as complete blockages [53], would be difficult if not impossible to detect microscopically. Therefore, we were unable to determine if blocked fleas were associated with the transmission events we observed. However, we recorded the presence of fleas that appeared to be clearly blocked (e.g., presence of fresh blood only in or anterior to the proventriculus) infrequently during our study (<3%), such that we believe the majority of the fleas used in our study were probably unblocked fleas.

All animal procedures in our experiments were approved by the Division of Vector-Borne Infectious Diseases (Centers for Disease Control and Prevention) Institutional Animal Care and Use Committee.

### Survival of fleas

For each time point, counts of total live and dead fleas at each temperature were recorded. The surviving fleas not used in the challenge feeds, were offered a maintenance meal on naïve mice and returned to incubators as described above. More than 17,800 fleas were infected in 12 separate artificial feeding events in our experiment. All of these fleas were included in evaluating the effects of temperature and time on flea survival in our analyses.

### Estimation of flea infection prevalences and bacterial loads

The infection status and bacterial loads in the fed fleas used in challenge feedings were determined by grinding fleas in heart infusion broth (HIB) supplemented with 10% glycerol and performing serial dilutions of flea triturate, plated in duplicate on 6% sheep blood agar plates. In some cases (n = 137), the flea triturate became contaminated with other bacteria, prohibiting accurate *Y. pestis *colony counts. In these cases, flea triturate dilutions were plated on selective *Yersinia *agar: Cefsulodin-Irgasan-Novobiocin base/HIB (CIN/HIB) agar. Colony counts of uncontaminated test samples on the CIN/HIB and sheep blood agar were of the same log, and therefore, colony counts for contaminated samples on CIN/HIB were not adjusted prior to analysis.

### Statistical analyses and estimation of per flea transmission efficiencies

Because we were primarily interested in the effects of warm temperatures on transmission and *Y. pestis in vivo *growth dynamics in fleas, and because we were only able to examine the effect of 10°C through day 14 p.i., we focused our temporal data analyses on comparisons among the three higher temperatures: 23, 27, and 30°C; therefore, unless otherwise indicated all tests discussed below excluded data from the 10°C treatment.

Transmission efficiencies per individual flea were estimated for each time point at each of the four temperature treatments, using maximum likelihood estimates calculated in the Microsoft Excel Add-In PooledInfRate, version 3.0 [58]. These estimates are based on the number of infected fleas that fed on an individual mouse and whether or not transmission was observed in that mouse.

The proportions of fleas surviving between time points were compared across temperatures and time points using quasibinomial regression analyses to adjust for the high degree of overdispersion in the proportion data. We examined the effects of temperature and days p.i. on the log_10_-transformed bacterial counts in fed fleas using a Poisson generalized linear model. Contingency table analyses were used to compare the proportions of fleas that successfully fed on naïve mice at each temperature and days p.i. and the effect of flea sex on feeding success across temperatures and days p.i. Contingency tables were also used to examine the effect of temperature on infection prevalences across days p.i.

We conducted a stepwise logistic regression analysis to identify the significant factors associated with the probability of *Y. pestis *transmission in our experiment. The explanatory variables considered in this analysis were temperature, time point, and variables that related to the characteristics of the flea group that fed on the mice, namely, the proportion of fed, infected fleas, the proportion of fed, infected female fleas, the summed bacterial loads of fed fleas, and the presence or absence of at least one fed flea harboring a minimum of 10^6 ^bacteria. The latter variable was considered because Hinnebusch et al. [59] and Engelthaler et al. [27] reported evidence that a bacterial load of at least 10^6 ^in a flea was required for blockage and transmission by *X. cheopis*.

### Effects of Y. pestis infection on flea survival at 10°C

To determine if the poor survival of fleas at 10°C was associated with the low temperature or infection by *Y. pestis*, a separate experiment was conducted in which fleas were assigned to an infected or non-infected treatment group, and thus were offered either a blood meal containing or not containing *Y. pestis*, respectively, during the artificial feed. The fed fleas (n = 569 infected and 576 non-infected) from these treatments were then placed in the incubator set at 10°C and monitored for survival on days 3, 7, 10, and 14. Any surviving fleas on these days were offered a maintenance blood meal on naïve Swiss Webster mice at room temperature as was done in our temperature experiment. The proportion of fleas surviving between time points was compared between the infected and uninfected groups using a quasibinomial regression analysis.

## Competing interests

The authors declare that they have no competing interests.

## Authors' contributions

AMS, RJE, KLG conceived and designed experiments; AMS, SWB, JLH, SMV, JAM, SKW, CBG, MEW performed experiments; AMS conducted data analysis; AMS, RJE, KLG, SWB interpreted results; AMS wrote paper. All authors read and approved the final manuscript.
